# Myelination of the right parahippocampal cingulum is associated with physical activity in young healthy adults

**DOI:** 10.1007/s00429-016-1183-3

**Published:** 2016-01-19

**Authors:** Tobias Bracht, Derek K. Jones, Sonya Bells, Sebastian Walther, Mark Drakesmith, David Linden

**Affiliations:** 1 Cardiff University Brain Research Imaging Centre (CUBRIC), School of Psychology, Cardiff University, Cardiff, UK; 2 Neuroscience and Mental Health Research Institute (NMHRI), Cardiff University, Cardiff, UK; 3 Translational Research Centre, University Hospital of Psychiatry, University of Bern, Bolligenstrasse 111, 3000 Bern, Switzerland; 4 MRC Centre for Neuropsychiatry Genetics and Genomics, School of Medicine, Cardiff University, Cardiff, UK

**Keywords:** White matter, Diffusion tensor imaging, Actigraphy, Activity, Hippocampus, Neuroplasticity

## Abstract

Recent evidence suggests that individual differences in physical activity (PA) may be associated with individual differences in white matter microstructure and with grey matter volume of the hippocampus. Therefore, this study investigated the association between PA and white matter microstructure of pathways connecting to the hippocampus. A total of 33 young, healthy adults underwent magnetic resonance imaging (MRI). High angular resolution diffusion-weighted imaging and multi-component relaxometry MRI scans (multi-component driven equilibrium pulse observation of T1 and T2) were acquired for each participant. Activity levels (AL) of participants were calculated from 72-h actigraphy recordings. Tractography using the damped Richardson Lucy algorithm was used to reconstruct the fornix and bilateral parahippocampal cinguli (PHC). The mean fractional anisotropy (FA) and the myelin water fraction (MWF), a putative marker of myelination, were determined for each pathway. A positive correlation between both AL and FA and between AL and MWF were hypothesized for the three pathways. There was a selective positive correlation between AL and MWF in the right PHC (*r* = 0.482, *p* = 0.007). Thus, our results provide initial in vivo evidence for an association between myelination of the right PHC and PA in young healthy adults. Our results suggest that MWF may not only be more specific, but also more sensitive than FA to detect white matter microstructural alterations. If PA was to induce structural plasticity of the right PHC this may contribute to reverse structural alterations of the right PHC in neuropsychiatric disorder with hippocampal pathologies.

## Introduction

### Physical activity and white matter microstructure

The benefits of physical activity (PA) for physical and mental health are well established (Hillman et al. 2008; Soundy et al. 2015). Meta-analyses in children suggest that higher PA is associated with improved cognitive performance (Sibley and Etnier 2003; Fedewa and Ahn 2011). For instance, a cross-sectional study found that higher PA is associated with better performance in executive functioning in children (van der Niet et al. 2014). PA has also been consistently reported to induce pleasurable affective responses (Ekkekakis et al. 2005; Bartlett et al. 2011). Cognitive performance and pleasure are also linked to brain structure (Fields 2008; Johansen-Berg 2010; Bracht et al. 2015a). Consequently, there has been an increasing interest in the effect of PA on the structure of the brain.

Diffusion tensor magnetic resonance imaging (DT-MRI) enables a non-invasive in vivo assessment of brain white matter microstructure (Basser et al. 1994). Diffusion properties such as fractional anisotropy (FA) indirectly reflect the extent to which the diffusion of water molecules in the brain tissue is preferentially hindered along one direction compared to others, which in turn provides information on the underlying white matter microstructure (Basser and Pierpaoli 1996). A previous DT-MRI study found a positive correlation between aerobic fitness and FA in the uncinate fasciculus and cingulum bundle (Marks et al. 2007). Findings in segments of the middle cingulum were replicated by the same group using objective markers of aerobic fitness (Marks et al. 2011). Walther et al. (2010), using 24 h recordings of actigraphy as an objective measure of motor activity to assess PA, found a positive association between activity levels (AL) and FA in the right superior longitudinal fasciculus (SLF) and cingulum bundle, and a negative correlation between AL and FA in the left corticobulbar tract, right posterior corpus callosum and left SLF (Walther et al. 2010). Further, associations between PA and white matter microstructure were identified in the corticospinal tract (Herting et al. 2014), the corpus callosum (Johnson et al. 2012) and in prefrontal, parietal and temporal brain regions (Voss et al. 2013). Thus, there is converging evidence that differences in PA are associated with differences in white matter microstructure in multiple brain regions.

### Hippocampal pathways, physical activity and clinical implications

In addition to brain regions mentioned above, the hippocampus is a further region of particular interest regarding the structural correlates of PA. Animal research suggests strongly that PA induces neuroplastic processes in the hippocampus. For example, it has been shown that wheel-running in mice increases cell proliferation in the dentate gyrus (van Praag et al. 1999a, b, 2005), and induces increases in brain-derived neurotrophic factor (BDNF), which supports survival of neurons, localized in hippocampal areas (Berchtold et al. 2005; Neeper et al. 1995). BDNF has been also shown to induce myelination in white matter pathways in both animal and in in vitro studies (Wong et al. 2014; Xiao et al. 2010). In elderly humans, PA was associated with increased volumes in bilateral hippocampi (Erickson et al. 2009). This finding was corroborated by findings that a PA exercise program (in contrast to a stretching program) led to increases in volume in the hippocampus (Erickson et al. 2011). Moreover, mean diffusivity (MD) has been shown to be reduced in elderly master athletes in a region incorporated in the parahippocampal cingulum in comparison with a less fit age-matched control group (Tseng et al. 2013), while a reduction in FA was reported in sedentary older adults in a recent DTI study (Burzynska et al. 2014).

Structural alterations of the hippocampus may not only be related to PA. Additionally, structural alterations of the hippocampus and its connection pathways have been reported consistently in neuropsychiatric disorders such as depression (Campbell and MacQueen 2004), schizophrenia (Heckers 2001) or dementia (den Heijer et al. 2010). Interestingly, PA may improve symptoms in aforementioned neuropsychiatric disorders (Mead et al. 2009; Heyn et al. 2004; Rosenbaum et al. 2014; Malchow et al. 2015). Thus there may be an association between PA, structural remodelling of hippocampal pathways and clinical recovery. This assumption is supported by a longitudinal study in major depressive disorder (MDD) identifying age-dependent white matter microstructural changes to occur in the left PHC during the time-span from depression to remission in a sample that also significantly increased their AL (Bracht et al. 2015b). In this study we exclusively focus on the pathways connecting to the hippocampus because these pathways may not only be associated with PA but PA may also impact on structural alterations in neuropsychiatric disorders such as schizophrenia, depression or dementia thereby potentially reversing structural alterations and leading to clinical improvements.

### Diffusion properties and myelin water fraction

Most previous studies using DT-MRI-based diffusion properties such as FA for the assessment of white matter microstructure are limited by the lack of specificity for white matter sub-compartments. For instance, increases in FA may stem from reductions in axonal diameter, higher axonal density, higher myelination, and/or lower intra-voxel orientational dispersion (Beaulieu 2002; Jones et al. 2013b). Thus, DT-MRI-based measures on their own do not allow for a sub-compartment specific, and therefore neurobiologically meaningful, interpretation of the data. Moreover, the lack of specificity may lead to conflicting results across studies. For instance, while greater myelination and larger axonal diameter both increase conduction velocity, they have opposite effects on FA.

The development of multicomponent driven equilibrium single pulse observation of T1 and T2 (McDESPOT) allows for rapid acquisition of data to produce whole brain myelin water fraction (MWF) maps which have been shown to correlate with myelination (Deoni et al. 2008a; Hurley et al. 2010; Laule et al. 2006; Moore et al. 2000; MacKay et al. 1994). One previous study used MWF as a marker of myelination and found a spatial pattern of myelination in infants that showed striking similarities with what is known from post-mortem studies (Deoni et al. 2011). In addition, studies in demyelinating neurological disorders such as multiple sclerosis strongly suggest the validity of MWF as a measure for myelination (Kolind et al. 2012; Kitzler et al. 2012). In one longitudinal study during early childhood changes of MWF correlated positively with performance measures such as gross motor behaviour, visual reception and receptive language (Dean et al. 2014). Finally, the most compelling evidence for the assumption that MWF indeed measures myelin stems from comparisons between MWF maps derived from a shaking pup myelin mutant and control animals (Hurley et al. 2010) and imaging studies demonstrating correlations between MWF and histopathology in multiple sclerosis (Laule et al. 2006; Moore et al. 2000). Consequently, there is ample evidence that MWF does correlate with myelination, suggesting that this measure may represent a useful marker to detect associations of brain structure and function. Therefore, the measure of MWF represents a significant step forward for the interpretability of previously reported changes in white matter, compared to reliance on DT-MRI alone.

### Aims of the study and hypothesis

The present study explored the association between PA and white matter microstructure of the two main hippocampal pathways: the fornix and the PHC. The fornix is the main efferent pathway of the hippocampus projecting to the mammillary bodies (Nieuwenhuys et al. 2007). The PHC forms part of the cingulum bundle and contains predominantly afferent projections from the posterior parietal cortex (Goldman-Rakic et al. 1984; Mufson and Pandya 1984; Jones et al. 2013a). In the present study we use FA as a well-established (though unspecific) marker of white matter microstructure. Further, we use MWF as a potentially more specific measure of myelination.

We hypothesize a positive correlation between FA and AL, and a positive correlation between MWF and AL, in the fornix and in bilateral PHC. We assume that due to the specificity for myelination, i.e., being less susceptible to confounding microstructural differences such as axonal diameter, density and axonal orientational dispersion than DT-MRI metrics, (De Santis et al. 2014) MWF will be a more sensitive marker than FA to detect associations between individual differences between PA and white matter microstructure.

## Methods

### Participants

All participants were recruited through the School of Psychology, Cardiff, Wales, UK. All participants were undergoing or had previously completed a university degree course, were right handed as assessed with the Edinburgh Handedness Inventory (Oldfield 1971) and of Caucasian origin. Exclusion criteria were a current episode or a history of neurological and psychiatric disorders, drug or alcohol abuse and medication that may have an impact on the structure of the brain. For assessment, the general health questionnaire was used (Goldberg and Huxley 1980). Since training may impact the structure of the brain we also excluded professional athletes, musicians and those at competitive amateur sport levels (Scholz et al. 2009; Bengtsson et al. 2005; Hanggi et al. 2010). A total of thirty-three participants was recruited (19 female, 14 male). Participants had a mean age of 25.5 ± 4.2 years. All participants provided written informed consent in order to take part in the study and received monetary compensation. The study had been approved by the local ethics committee of the School of Psychology, Cardiff (EC.13.07.02.3491RA).

### Actigraphy

Participants wore an Actiwatch (Cambridge Neurotechnology, Inc., Cambridge, UK) on the left wrist for 72 consecutive hours. Activity counts were stored in 5 s intervals. Participants provided an activity protocol stating the kind of daily activities and wake time. Activity levels (AL) (the cumulated activity counts during wake time divided by the net recording time in hours) were calculated separately for each day. Activity analyses were restricted to wake time. Mean-AL was calculated by averaging AL over the three consecutive days. The left wrist was chosen because AL of the non-dominant arm reflects whole body movements without impact of manual fine motor activities (Middelkoop et al. 1997). Protocols were checked for consistency between reported activities and AL measures. Almost identical approaches have been repeatedly used in previous studies e.g. (Bracht et al. 2012; Walther et al. 2009; Razavi et al. 2011).

### Structural MRI scanning

T_1_-weighted structural scans were acquired using an oblique axial, 3D fast-spoiled gradient recalled sequence (FSPGR) with the following parameters: TR = 7.9 ms, TE = 3.0 ms, inversion time = 450 ms, flip angle = 20°, 1 mm isotropic resolution, with a total acquisition time of approximately 7 min.

### Diffusion MRI scanning

High angular resolution diffusion-weighted imaging (HARDI) data were acquired in the Cardiff University Brain Research Imaging Centre (CUBRIC) on a 3 T GE Signa HDx system (General Electric, Milwaukee, USA) using a cardiac-gated peripherally gated twice-refocused spin-echo Echo Planar Imaging (EPI) sequence, with effective TR/TE of 15R-R intervals/87 ms. Sets of 60 contiguous 2.4 mm thick axial slices were obtained, with diffusion-sensitizing gradients applied along 30 isotropically distributed (Jones et al. 1999) gradient directions (*b* value = 1200 s/mm^2^). The field of view was 23 × 23 cm; and the acquisition matrix was 96 × 96, resulting in data acquired with a 2.4 × 2.4 × 2.4 mm isotropic resolution. Following zero-filling to a 128 × 128 in-plane matrix for the fast Fourier transform, the final image resolution was 1.8 × 1.8 × 2.4 mm. A parallel acceleration (ASSET) factor of 2 was used. Acquisition time was approximately 12 min.

### McDESPOT scanning

The McDESPOT protocol consists of a combination of sagittally oriented spoiled gradient recalled (SPGR), balanced steady-state free procession (bSSFP) and inversion-recovery prepared SPGR (IR-SPGR) sequences (Deoni et al. 2008c, d). All three sequences were acquired with a FOV of 220 mm; 1.7 mm × 1.7 mm × 1.7 mm voxels, with frequency encoding in the superior-inferior direction for a total scan time of approximately 12 min. mcDESPOT protocol: SPGR, acquisitions: TE = 2.1 ms, TR = 4.7 ms, flip angles = [3°, 4°, 5°, 6°, 7°, 9°,13°, 18°]; bSSFP acquisitions: TE = 1.6 ms, TR = 3.2 ms, flip angles = [10.6°, 14.1°, 18.5°, 23.8°, 29.1°, 35.3°, 45°, 60°]. bSSFP acquisitions were repeated with and without 180° RF phase alteration to remove SSFP banding artefacts and SPGR and IR-SPGR acquisitions were used to correct B0 and B1-induced errors in the derived MWF estimates.

### McDESPOT data pre-processing

SPGR and bSSFP images for each participant were linearly coregistered using an affine (12 degrees of freedom) technique based on mutual information to the first image in the sequence to correct for interscan and intrascan motion (Jenkinson and Smith 2001). SPGR and IR-SPGR images were used for DESPOT1 with High-speed Incorporation (DESPOT1-HIFI) of RF Field Inhomogeneities processing as described in (Deoni et al. 2006b; Deoni 2007), resulting in B_1_ field and quantitative T_1_ maps. These B_1_ field and T_1_ maps were used in the subsequent calculation of B_0_ field and T_2_ maps using two phase-cycled bSSFP data using the DESPOT2 with full modeling (DESPOT2-FM) algorithm (Deoni et al. 2004). Combining SPGR, IR-SPGR and SSFP sequences allowed for the estimation of the multi-component three pool DESPOT model (Deoni et al. 2008b, c, 2013). Alongside other metrics not studied here (myelin water residence time and intra- and extra-cellular (IE) water and myelin water T_1_ and T_2_), this model provides whole brain estimates of the myelin water fraction (MWF). R_1_ maps were computed by taking the reciprocal of T_1_.

### Post-processing of McDESPOT data

A synthetic-T_1_ image was computed from the quantitative T_1_ map (arising from the DESPOT1 data) for each subject assuming the same imaging parameters used to generate the FSPGR T1-weighted image (Deoni et al. 2006a). This effectively creates a template in MWF space with the same contrast as the target T_1_-weighted image. The synthetic T_1_-weighted image of each participant was then warped to the corresponding T_1_-weighted structural scan using the FNIRT non-linear registration tool (Jenkinson et al. 2002). The computed warps were then applied to the MWF map and to the quantitative T_1_ map (which is being used to calculate R_1_) to transform those maps into the same space as the structural T_1_-weighted image.

### Diffusion MRI data pre-processing

Data were analysed using *ExploreDTI* *4.8.3* (Leemans et al. 2009). Eddy-current induced distortion and motion correction was performed using an affine registration to the non-diffusion-weighted B_0_-images, with appropriate re-orienting of the encoding vectors (Leemans and Jones 2009). Field inhomogeneities were corrected for using the approach of (Wu et al. 2008). The diffusion weighted images (DWIs) were non-linearly warped to the T_1_-weighted image using the FA map, calculated from the DWIs, as a reference. Warps were computed using Elastix (Klein et al. 2010) using normalized mutual information as the cost function and constraining deformations to the phase-encoding direction. The corrected DWIs were therefore transformed to the same (undistorted) space as the T_1_-weighted structural images. A single diffusion tensor model was fitted to the diffusion data in order to compute quantitative parameters such as FA (Basser et al. 1994). Following the method of Pasternak et al. (Metzler-Baddeley et al. 2012; Pasternak et al. 2009) a correction for free water contamination of the diffusion tensor based estimates was applied (Metzler-Baddeley et al. 2012; Pasternak et al. 2009). Data quality was checked by careful visual inspection and by looking at the average residuals per DWI for each participant.

### Tractography

Tractography was performed using *ExploreDTI* (Leemans et al. 2009). Whole brain deterministic tractography was performed following peaks in the fibre orientation density function (fODF) reconstructed from the damped Richardson Lucy algorithm (dRL) (Dell’acqua et al. 2010; Jeurissen et al. 2013). The dRL algorithm estimates multiple fibre orientations in a single voxel and therefore provides a more accurate diffusion profile than it is the case for DT-MRI-based methods estimating only one fibre orientation per voxel. For each voxel in the dataset, streamlines were initiated along any peak in the fibre orientation density function (fODF) that exceeded an amplitude of 0.05. A streamline, uniform step-size, algorithm based on that of (Basser et al. 2000), but extended to multiple fibre orientations within each voxel (Jeurissen et al. 2011) was used for tractography. Each streamline continued in 0.5 mm steps following the peak in the fODF that subtended the smallest angle to the incoming trajectory. Termination criteria were an angle threshold >45° and fODF amplitude <0.05.

### Tract reconstruction

**Fig. 1:**
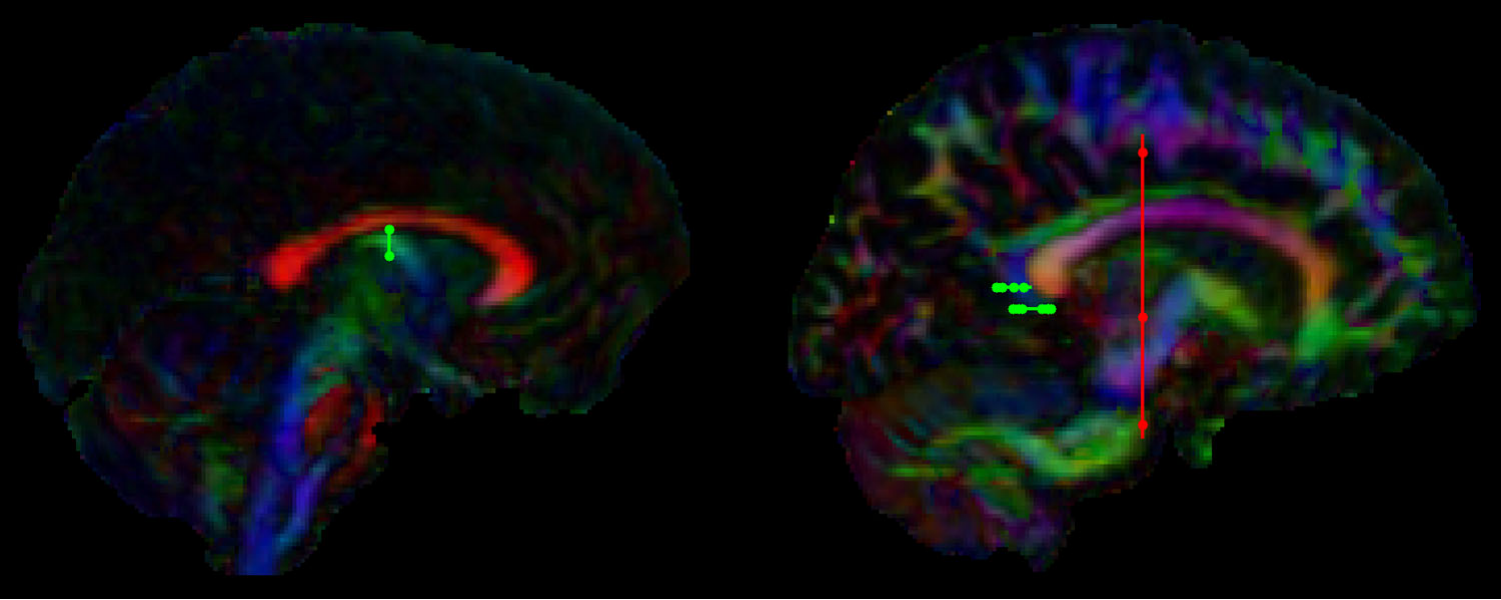
Tract “waypoint” regions for the fornix (*left*) and the parahippocampal cingulum (*right*) are visualized in *green*, “NOT” regions are visualized in *red*

**Fig. 2:**
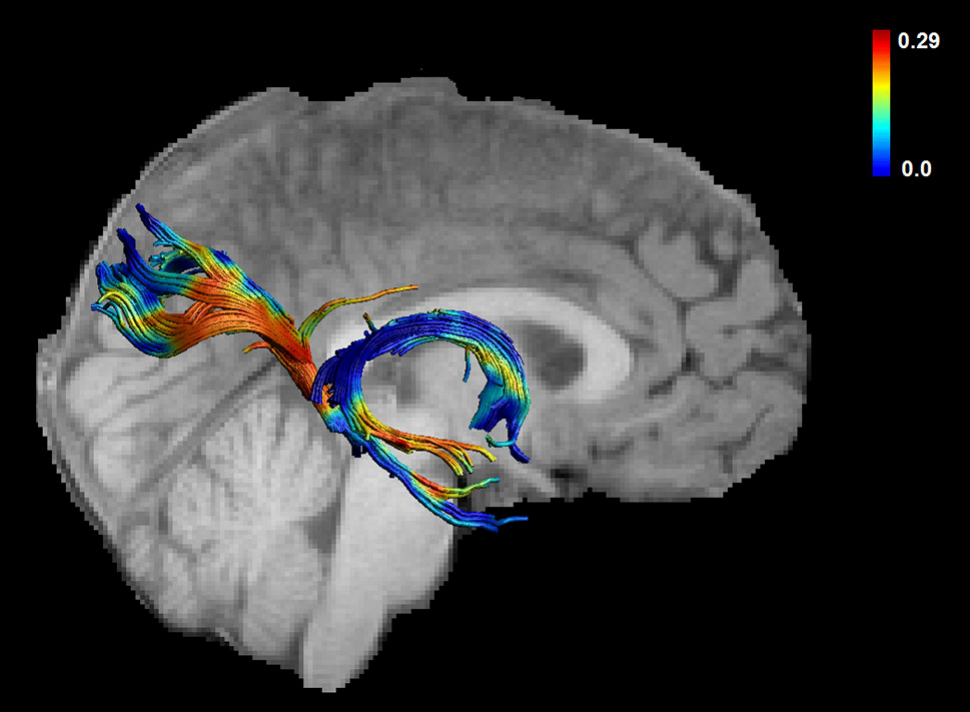
Myelin water fraction sampled across the fornix and the PHC for one representative subject

Tract ‘waypoint’ regions were drawn manually by one experimenter (T.B.) based on anatomical landmarks. The fornix and bilateral PHC (see Figs. 1, 2) were reconstructed according to previously described algorithms (Bracht et al. 2015b; Metzler-Baddeley et al. 2013; Jones et al. 2013a). For reconstruction of the fornix, a coronal region of interest (ROI) was placed around the columns of the fornix four slices posterior to the anterior commissure (Bracht et al. 2015b). For reconstruction of bilateral PHC one horizontal ROI was placed at the height of the most ventral point of the splenium, and a second ROI was placed four slices above. A NOT ROI was placed above the body of the corpus callosum caudal to the rostral-caudal midpoint of the body of the corpus callosum as described in (Metzler-Baddeley et al. 2013; Jones et al. 2013a). For each subject, the anatomical course of each tract was checked carefully. Mean-FA was derived for each reconstructed tract. In addition, the average mean diffusivity (MD) and axial and radial diffusivity (AD, RD) were computed for each tract. Further, mean-MWF and R1 were sampled along the tracts. Those were derived from the MWF-and the single T1-image respectively that had been warped to the T_1_-weighted structural image.

### Statistical analyses

Statistical analyses were performed using SPSS22 (SPSS, Inc., Chicago, IL, USA). First, normal distribution of mean-AL, FA and MWF-values was confirmed using Shapiro–Wilk-Tests. Second, Pearson correlations between the mean-AL and FA of bilateral PHC and the fornix were calculated. Third, correlations between mean-AL and MWF, our measure for myelination, were calculated for each of the three hippocampal pathways. We applied a strict Bonferroni correction for multiple comparisons. Thus the level of significance was set at *p* < 0.0083 (0.05 divided by the number of tests, *n* = 6). In pathways where significant correlations were detected we additionally controlled for age and gender calculating separate partial correlation with age and gender as covariates. Further, significant correlations between AL and FA were followed up using MD, RD and AD. Significant correlations between AL and MWF were followed up using R1.

## Results

### Activity levels

One participant had to be excluded from the analyses because their actigraphy recording was incomplete and there had been inconsistencies between reported activities and recorded AL. Thus a total of 32 participants with AL-recordings remained. AL was normally distributed for each of the three single days and for mean-AL (averaged over the 3 days). Repeated measure analyses revealed no significant differences of AL for the three consecutive days [*F*(2, 30) = 0.37, *p* = 0.70]. AL-values were as follows: AL day 1 = 20,513 ± 6081, AL day 2 = 21,496 ± 8000, AL day 3 = 21,516 ± 6174, and mean-AL = 21,174 ± 5345. Men and women did not differ regarding AL for any of the days or regarding mean-AL.

### Correlations between activity levels and white matter microstructure

For each of the tracts, FA and MWF values were normally distributed. Across the 32 participants there were no significant correlations between mean-AL and FA for the fornix (*r* = 0.232, *p* = 0.201), left (*r* = −0.123, *p* = 0.503) or right (*r* = 0.047, *p* = 0.800) PHC.

**Fig. 3:**
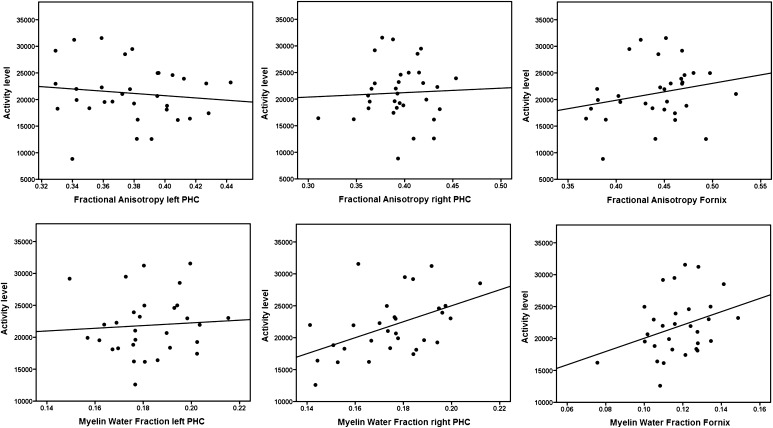
Correlations between activity levels and fractional anisotropy and between activity levels and myelin water fraction are shown for the left and the right parahippocampal cingulum (PHC) and the fornix

Two participants had to be excluded from the McDESPOT analyses because of incomplete data acquisition, and thus 30 scans remained. There was a positive correlation between mean-AL and MWF for the right (*r* = 0.482, *p* = 0.007) but not for the left (*r* = 0.069, *p* = 0.718) PHC (see Fig. 3). Further, there was a non-significant trend for a positive correlation between MWF of the fornix and mean-AL (*r* = 0.325, *p* = 0.079). The significant correlation between AL and MWF of the right PHC was followed up by calculating correlations between AL and R1 of the right PHC. A non-significant trend was found (*r* = 0.357, *p* = 0.053). The correlation between the right PHC and mean-AL remained significant after controlling for age (*r* = 0.531, *p* = 0.003) and for gender (*r* = 0.483, *p* = 0.008). In order to statistically demonstrate the larger magnitude of the correlation between AL and MWF of the right PHC in comparison to the correlation between AL and FA of the right PHC Fisher’s r-to-z transformation was used (*z* = 3,6, *p* = 0.0003) (Steiger 1980) (http://quantpsy.org/corrtest/corrtest2.htm).

## Discussion

This is the first tractography study linking white matter microstructure of hippocampal pathways to PA. This is also the first study investigating the association between PA and MWF, a more specific measure of myelination than FA. While correlations between FA and AL did not yield significant results for any of the tracts, there was a significant correlation between AL and MWF for the right PHC. Thus, our results suggest that higher PA is associated with higher myelination in the right PHC. We infer, therefore, that MWF not only represents a more specific marker for myelination but is also a more sensitive marker than FA for detecting associations between white matter microstructure and PA. Our results contribute to the understanding of brain-behaviour associations and inter-individual variance even in a highly homogeneous group of young adults.

### Myelination of hippocampal pathways and physical activity

In our study we found a selective positive correlation between PA and MWF in the right PHC. Further, there was a non-significant trend (*r* = 0.325, *p* = 0.079) for a positive correlation between PA and the fornix. Given that the MWF is a putative marker of myelin our findings suggest that higher PA is reflected by higher myelination of the right PHC. Based on the cross-sectional study design it remains unclear if increases in myelination of the right PHC are cause or consequence of increased PA. However, histological studies in animals clearly suggest that experience can influence the degree of myelination. For instance, stress during pregnancy in rodents, the degree of social interactions and the number of play objects all lead to increases myelination (Bennett et al. 1964; Szeligo and Leblond 1977; Markham and Greenough 2004; Wiggins and Gottesfeld 1986). Moreover, electrical stimulation of the premotor cortex in mice has been shown to cause increases in myelination which were associated with improved motor function of the corresponding limb (Gibson et al. 2014). In vitro studies also suggest that neuronal activity by means of neurotransmitter release promotes myelin induction (Demerens et al. 1996; Wake et al. 2011). Further, there is converging evidence from longitudinal neuroimaging studies in humans that PA increases grey matter volume of the hippocampus (Erickson et al. 2011; Pajonk et al. 2010) which may impact plasticity of white matter microstructure as well. Thus, while our study design does not allow for statements regarding causalities of the observed association between MWF and AL, multiple lines of evidence suggest that experience and behaviour indeed induce remodelling of myelination of the brain (Fields 2008).

### Comparison with previous DTI studies

We did not find any associations between PA and FA in bilateral PHC and in the fornix. Thus in our sample putative changes in myelin could not be detected using FA. While DT-MRI metrics can be sensitive to differences in myelin (Song et al. 2002) it is worth noting that in genetically modified mice in which myelin has very little presence (e.g. the Shiverer mouse), FA is only lowered around 15 % compared to a wild type mouse (Song et al. 2002). Consequently, changes in myelin of a few percent would have a very small impact on FA. Conversely, as measures such as MWF are thought to be more directly associated with myelin, their sensitivity to myelin changes should be more marked. This may explain why we found a significant correlation between PA and MWF but not between PA and FA.

The absence of a correlation between FA und PA is in line with a voxel-based DT-MRI study of (Walther et al. 2012) who found no association between AL and FA in healthy controls in the PHC. However, there was a negative association of FA and AL in MDD in the left PHC (Walther et al. 2012). A whole brain tract-based spatial statistics (TBSS) (Smith et al. 2006) study found reduced mean diffusivity (MD) in physically fit older adults compared to a less fit control group in a region incorporating the PHC localized in the left hemisphere as well (Tseng et al. 2013). However, PA was not associated with FA. On the other hand (Burzynska et al. 2014) reported a decrease of FA in sedentary old adults averaged across bilateral PHC using TBSS.

Thus, findings are inconsistent regarding identified diffusion properties (e.g. FA and MD), lateralization and directionality of associations between PA and diffusion properties. In part, this may be owed to differences in study populations. It is possible that in different populations (e.g. students, physically fit or sedentary populations, clinical populations) associations between brain structure and function are reflected by changes in different sub-compartments of white matter microstructure (e.g. changes in axonal diameter, density or myelination) that may in aggregate have different effects on different DT-MRI-based indices (such as FA and MD). However, due to the lack of specificity of DT-MRI-based metrics for white matter sub-compartments this cannot be disentangled. Therefore, the myelin specific measure of MWF represents a significant step forward for the interpretability of white matter neuroimaging studies. A further explanation for those discrepancies is that age impacts on white matter microstructure (Lebel et al. 2010) and neuroplasticity of the PHC (Bracht et al. 2015b). Thus, it is possible that in our young sample changes predominantly occur in myelination which only has a subtle effect on FA (Song et al. 2002), while findings in diffusion properties in elderly populations may be the result of different neuroplastic processes (e.g. neuroplasticity of the axons) (Tseng et al. 2013; Burzynska et al. 2014). Moreover, contrasting results between studies may be owed to differences in methodological approaches. For instance, tractography approaches may be more sensitive than voxel-based analyses or regions of interest (ROI)-analyses to detect tract-specific group differences (Bracht et al. 2014, 2015a; Keedwell et al. 2012; Kanaan et al. 2006).

### Lateralization

Our specific finding of an association between PA and MWF of the PHC for the right hemisphere in young students complements findings of associations between PA and diffusion properties (FA and MD) for the left hemisphere in MDD and in an elderly population (Tseng et al. 2013; Walther et al. 2012). Since in contrast to MWF DT-MRI-based diffusion properties are not specific for myelin and completely different populations have been investigated using different methodological approaches comparability of the latter findings (Tseng et al. 2013; Walther et al. 2012) with our study in young and healthy participants is limited. It is possible that if those groups had used tractography and looked at MWF as well, a right-sided association between PA and MWF of the PHC would have been identified. Clearly, studies investigating separate homogeneous subgroups with comparable methodological approaches are called for to reliably address the question of differences in lateralization in terms of associations between white matter microstructure and PA.

### Clinical implications

Assuming that PA may indeed induce neuroplastic processes in hippocampal pathways our finding is also of clinical importance. Reduced PA is a clinical feature in various neuropsychiatric disorders with putative hippocampal changes including depression and schizophrenia (Walther 2015; Schrijvers et al. 2008; Bracht et al. 2015c). There is some preliminary evidence that increasing PA (e.g. by means of exercise programs) may help alleviate clinical symptoms (Cooney et al. 2013; Wolff et al. 2011). One may speculate whether clinical improvements following exercise interventions in those disorders are caused by inducing neuroplastic structural changes in pathologically altered projection pathways of the hippocampus (Pajonk et al. 2010). For instance, as suggested by our finding increases in PA may induce myelination of the right PHC. Longitudinal clinical studies using tractography in conjunction with McDESPOT that investigate patient groups at baseline and following an exercise intervention program are required to address this question in future studies.

### Summary and conclusions

In this study we combined an advanced tract-specific approach (dRL) with a myelin specific measure that enables us to draw anatomically meaningful conclusions from our findings (Dell’acqua et al. 2010; Deoni et al. 2008a). A further strength of the study is the homogeneous sample which reduces much of the variance that otherwise is difficult to control for. Our findings suggest that higher PA is associated with higher myelination of the right PHC. Replication studies are called for to validate this initial finding. Given the non-significant trend for an association between PA and MWF of the fornix it would also be of interest to readdress this hypothesis in future studies.

Our results substantially extend previous findings on associations between PA and white matter microstructure. Most importantly, we provide a more myelin-specific measure that allows for a more neurobiologically meaningful interpretation of our data. Furthermore, we chose a tractography-based approach that (in contrast to automated voxel-based approaches) takes individual differences of the course of anatomical pathways into account, and combines multiple samples into the estimate, increasing the statistical sensitivity (Bells et al. 2011). Since the applied dRL-tractography algorithm estimates multiple fibre orientations within a single voxel, inaccuracies that lead to spurious reconstructions if applying DTI-based tractography can be diminished (Dell’acqua et al. 2010).

Since we investigated a homogeneous group of young students our findings may not generalize to a population of non-university students. Further, our study is limited by the cross-sectional study design, which does not allow us to determine whether PA influences white matter microstructure or whether white matter microstructure impacts on motor behaviour. Longitudinal studies are required to address this research question (Scholz et al. 2009). Furthermore, advanced white matter mapping techniques with specificity for axonal properties (e.g. density, diameter) may complement our myelin specific findings (Assaf and Basser 2005).
